# Limited genetic variability of *Cytauxzoon feli*s apical membrane antigen-1 (*ama1*) from domestic cats and bobcats

**DOI:** 10.1186/s13071-019-3347-5

**Published:** 2019-03-19

**Authors:** Jaime L. Tarigo, Lisa S. Kelly, Holly M. Brown, David S. Peterson

**Affiliations:** 1 Department of Pathology, College of Veterinary Medicine, University of Georgia, Athens, GA USA; 2 Metzger Animal Hospital, State College, PA USA; 3 Department of Infectious Diseases, Center for Tropical and Emerging Global Diseases University of Georgia, Athens, GA USA

**Keywords:** *Cytauxzoon felis*, Domestic cats, Bobcats, Genotypes, Genetic marker, Apical membrane antigen-1, Nucleotide diversity (π), Haplotype diversity (H), Polymerase chain reaction (PCR), Real-time PCR

## Abstract

**Background:**

*Cytauxzoon felis* is a tick-transmitted apicomplexan that causes cytauxzoonosis in domestic cats (*Felis catus*). Even with intensive care, the mortality rate of acute cytauxzoonosis approaches 40% in domestic cats, while bobcats (*Lynx rufus*), the natural intermediate host of *C. felis*, remain clinically asymptomatic. However, multiple reports of domestic cats surviving acute disease without any treatment exist. One hypothesis for survival of these cats is infection with unique *C. felis* genotypes of lower pathogenicity. Prior studies have identified genetically distinct *C. felis* isolates containing polymorphisms within internal transcribed spacer regions (ITS) of the rRNA operon. However, these polymorphisms do not correlate with the clinical outcome of cytauxzoonosis, and so additional genetic markers are needed to test this hypothesis. We selected *C. felis* apical membrane antigen-1 (*ama1*) as a potential genetic marker of differential pathogenicity. AMA1 is a vaccine candidate for relatives of *C. felis* within *Plasmodium* spp.; however its historically high level of genetic polymorphism has resulted in escape from vaccine-induced immunity. While such diversity has hindered vaccine development, the expected polymorphism within the *ama1* gene may be useful to evaluate population genetics.

**Results:**

A 677 bp sequence of the *C. felis ama1* gene was PCR-amplified from 84 domestic cats and 9 bobcats and demonstrated 99.9% sequence identity across all samples. A single nucleotide polymorphism (SNP) was identified in domestic cats and bobcats with evidence for co-infection with both genotypes identified in two domestic cats. The prevalence of the two genotypes varied with geographical distribution in domestic cats. Nucleotide diversity (π) and haplotype diversity (H) were calculated for *C. felis ama1* and *ama1* of related apicomplexans to assess genetic diversity. Based on these values (π = 0.00067 and H = 0.457), the diversity of the *C. felis ama1* gene region analyzed is considerably lower than what is documented in related apicomplexans.

**Conclusions:**

In surprising contrast to related apicomplexans, our results support that the sequence of the *C. felis ama1* gene is highly conserved. While lack of genetic diversity limits utility of *C. felis AMA1* as a genetic marker for clinical outcome, it supports further investigation as a vaccine candidate for cytauxzoonosis.

## Background

Cytauxzoonosis is a life-threatening infectious disease of domestic cats (*Felis catus*) caused by the tick-transmitted apicomplexan *Cytauxzoon felis*. A recent study found an overall prevalence rate for *C. felis* of 6.2% in three U.S. states, with over 15% in Arkansas alone, demonstrating relevance of infection to veterinarians [1]. The bobcat (*Lynx rufus*) is the natural host for *C. felis* and unlike domestic cats, infected animals are typically asymptomatic [2]. While there has been limited prevention available for cytauxzoonosis, recent studies have reported promising results for prevention of transmission of *C. felis* with the use of a combinatorial selamectin (6.0 mg/kg) plus sarolaner (1.0 mg/kg) topical product (Revolution® Plus, Zoetis) as well as with 10% imidacloprid/4.5% flumethrin collars (Seresto®, Bayer) [3, 4]. For domestic cats that presented to veterinary hospitals with acute disease, mortality rates were 40% and 74% when treated with supportive care and combined atovaquone and azithromycin or imidocarb respectively [5]. Interestingly, domestic cats surviving infection without any treatment have been reported [6, 7]. A study of cytauxzoonosis in northwestern Arkansas and northeastern Oklahoma indicated survival of natural *C. felis* infection in 18 cats with and without treatment [6]. The cats in this study were reported to experience milder clinical symptoms than those typically associated with *C. felis* infection. Similar sporadic reports in other areas exist [8]. One hypothesis for the striking variability of the clinical outcome of some cats with cytauxzoonosis is infection with unique *C. felis* genotypes of different pathogenicity.

Studies to date attempting to link specific genotypes to clinical outcome have focused on identifying polymorphisms within the first and second internal transcribed spacer regions of the rRNA operon (ITS1 and ITS2) [9–11]. These studies demonstrated genetically distinct *C. felis* populations geographically, and suggested a diverse population structure for *C. felis*; however, ITS polymorphisms were not associated with pathogenicity and clinical outcome. Additional genetic markers will be required to fully address questions of population genetics and, importantly, the possibility that particular *C. felis* genotypes are linked to clinical outcome in domestic cats [1, 9, 10, 12].

One candidate for identification of polymorphisms that may be associated with clinical outcome is the apical membrane antigen-1 (*ama1*) gene. AMA1 is a surface membrane protein unique to apicomplexan pathogens that allows for efficient invasion of host cells. Blockade of AMA1 *via* specific antibodies has been associated with inhibition of host cell invasion for several apicomplexans including *Plasmodium* spp. [13–15], *Babesia* spp. [16], *Toxoplasma gondii* [17] and *Theileria parva* [18]. As such, AMA1 has been subject to diversifying immunological pressure resulting in significant polymorphism in *Plasmodium* spp. [19–23] and a variable degree of polymorphism in *Babesia* spp. [24–26]. As a highly polymorphic antigen would have utility in defining *C. felis* population genetics, we investigated the *ama1* gene of *C. felis* for sequence variation and association with clinical outcome of infection in domestic cats, as well as geographical origin of infection in bobcats and domestic cats.

## Results

**Fig. 1:**
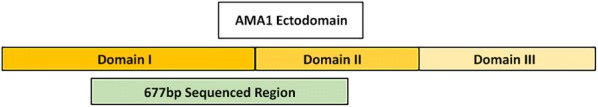
Sequenced region of *C. felis ama1* within domains I and II of the predicted ectodomain

Prior to the release of the *C. felis* genome sequence, the homology-based PCR approach employed was required to obtain the 677 bp region of the *C. felis ama1* gene evaluated in this study. This gene region codes for portions of the AMA1 domains I and II of an immunogenic ectodomain, which are important targets of inhibitory antibodies in *P. falciparum* AMA1 (Fig. 1) [27]. In addition, a study of *P. vivax ama1 gene* sequence diversity in samples from Sri Lanka found SNPs to be more prevalent in domains I and II than in domain III, suggesting that these regions would be a good source of genetic diversity in the homologous *C. felis* gene [21].

**Fig. 2:**
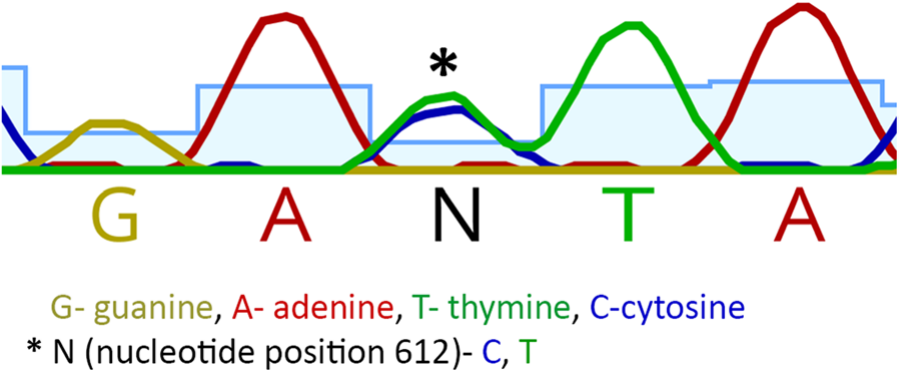
Coinfection with *C. felis* genotypes containing both SNPs (C, T) in two domestic cats from Georgia

Comparison of the 677 bp *C. felis ama1* PCR product amplified from 84 domestic cats and 9 bobcats demonstrated 99.9% sequence identity across all samples (GenBank: MH568694). A single nucleotide polymorphism (SNP) coding for a synonymous substitution (cytosine or thymine) at position 612 was identified (Table 1). Evidence for co-infection with genotypes containing both SNPs was identified in two domestic cats from Georgia (Fig. 2). An additional SNP, coding for a non-synonymous substitution (adenosine to thymine, encoding an Asn or Asp), was identified in one cat from Georgia at position 649. The prevalence of the two genotypes varied with geographical distribution (Chi square test, *χ*^2^ = 53.66, *df* = 1, *P *≤ 0.0001) in domestic cats with single-genotype infections. In Arkansas, the sequence from all 53 domestic cats contained a thymine at position 612, whereas in Georgia the presence of cytosine at this position was more common (Table 1); a similar trend of geographical distribution was observed in the 9 bobcat samples.

**Table 1:** Differential geographical distribution of *C. felis* AMA1 genotypes in domestic cats and bobcats

	Domestic cats			Bobcats		
	C	T	C/T	C	T	C/T
Arkansas^a^	0	53	0	0	2	0
California	0	0	0	0	1	0
Florida	0	1	0	5	1	0
Georgia^a^	21	7	2	3	0	0

^a^Chi-square test, *χ*^2^ = 53.66, *df* = 1, *P* ≤ 0.0001

*Abbreviations*: C, cytosine; T, thymine

**Fig. 3:**
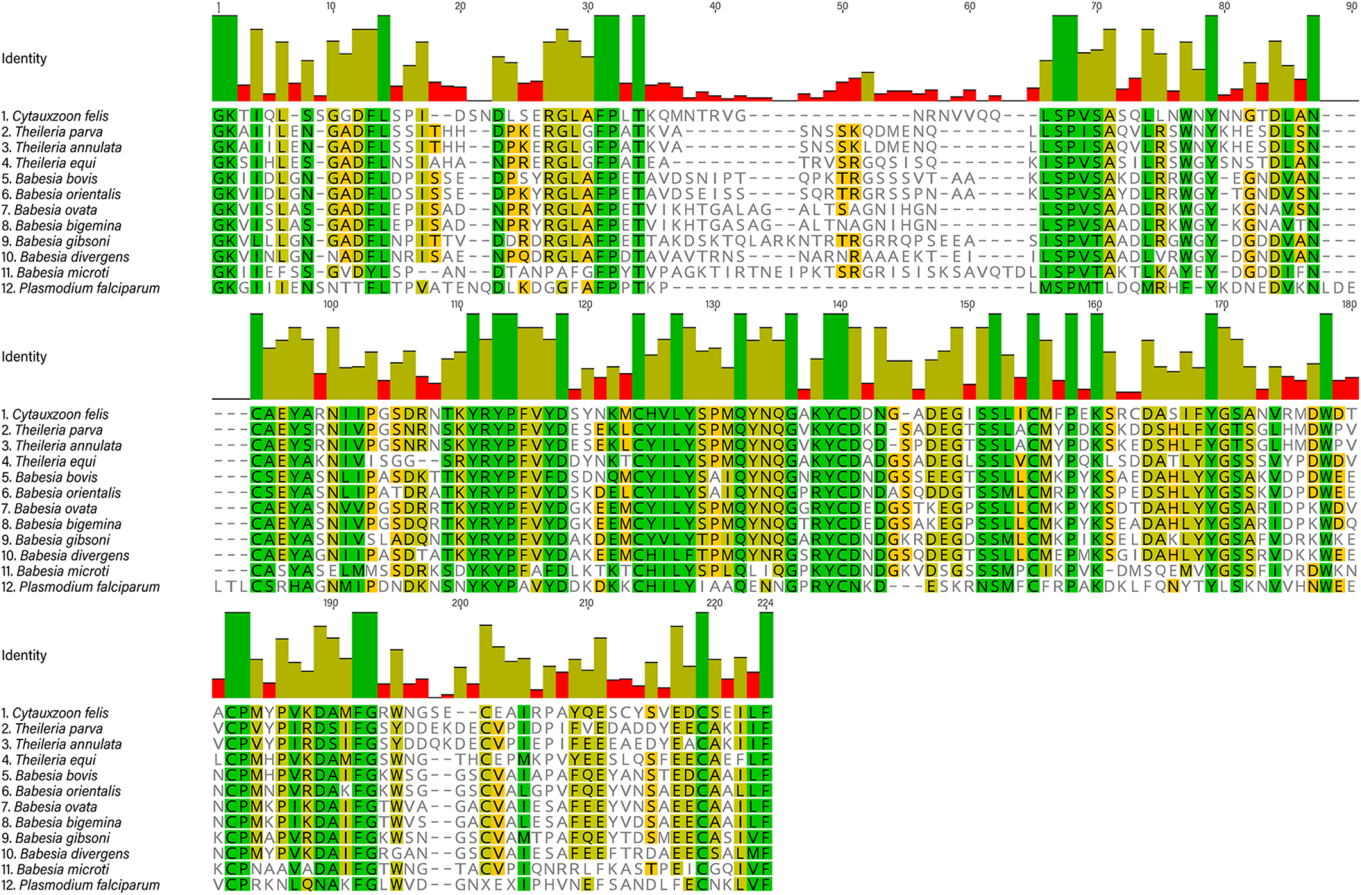
Amino acid sequences of *C. felis * AMA1 and related apicomplexans

Given the strikingly low level of observed sequence polymorphisms within our sample set, we sought to compare the degree of polymorphism in the *C. felis ama1* gene region with homologous published gene sequences in the related apicomplexan parasites *B. bovis*, *B. bigemina*, *T. annulata*, *P. vivax* and *P. falciparum.* We first used the translated sequences of the *C. felis ama1* gene region to construct an alignment with amino acid sequences from related apicomplexans. This was used to refine the nucleotide region for each species to be used in the genetic diversity calculations such that each analysis was done on the same relative region comprised of sequence within domain I and II of the AMA1 ectodomain (Fig. 3). We calculated two measures of genetic diversity for *ama1* from each species, which were nucleotide diversity (π) and haplotype diversity (H). Nucleotide diversity (π) is the average number of nucleotide differences per site in pairwise comparisons, and haplotype diversity (H) is the probability that two randomly chosen haplotypes will be different. The values of π and H for the *C. felis ama1* gene are considerably lower than those of related apicomplexans. While such measurements depend upon factors related to the population sampled, based upon available gene sequences in GenBank, the *C. felis ama1* gene region analyzed has a nucleotide diversity 15–30-fold lower than closely related parasites (Table 2).

**Table 2:** Nucleotide diversity (π) and haplotype diversity (H) within the *ama1* gene among apicomplexan parasites

Organism (no. of sequences)	π	H
*C. felis*^a^ (93)	0.00067	0.457
*T. annulata*^b^ (12)	0.01069	0.900
*B. bovis*^c^ (17)	0.02103	0.926
*B. bigemina*^d^ (29)	0.01104	0.995
*P. vivax*^e^ (23)	0.00986	0.941
*P. falciparum*^f^ (135)	0.01321	0.752

NCBI GenBank accessions:

^a^MH568694

^b^KX231669-680.1

^c^AB787632-37; AY486101; FJ588024-28; KX196261-64; XM_001610993

^d^AB481200; GQ257738-40; HM543726-30; JN572795-801; KP000032-33; KU557535/38; XM_012911354

^e^EF218679-701

^f^KT897327-461.1

## Discussion

There are several potential factors that may correlate with clinical outcome of *C. felis* infection. Hypotheses for survival in cats without treatment may include an atypical route of infection, innate immunity in certain cats, age or immune status, concurrent infections, decreased virulence with strain attenuation, lower dose of infectious inoculum, and inoculation by a less competent vector. In this study, we sought to test the hypothesis that *C. felis* strains with unique genotypes were associated with clinical outcome, and assessed the potential of the *ama1* gene as a useful marker to distinguish different parasite isolates. A region of the *ama1* gene of *C. felis* was investigated for sequence variation associated with clinical outcome of infection in domestic cats. In contrast to *Plasmodium* spp., the causative agents of malaria in people, our results support a highly conserved *C. felis ama1* gene sequence. One SNP was identified in a 677 bp region and given the synonymous nature of the substitution, it is unlikely to correlate with different clinical outcomes. The SNP, however, was significantly associated with geographical distribution of *C. felis* genotypes in domestic cats which may prove useful in conjunction with other markers for tracking future geographical patterns of this highly fatal disease.

AMA1 has been a major focus of vaccine development against human malarial parasites; however, successful vaccine development has been hampered by the presence of extensive polymorphism, with over 200 *P. falciparum* haplotypes found within samples from one village in Mali [28]. The lack of genetic diversity observed in the *C. felis ama1* gene strongly suggests that it is not under immune driven balancing selection as is the case for the homologous gene in *P. falciparum* [29]. There are several potential explanations for this striking lack of genetic diversity. It is possible that the host–parasite interactions within the bobcat host have affected a purifying selection on the *ama1* gene, such that very few haplotypes are now found. This could be due to lack of genetic diversity within the bobcat population, which may not have the diversity of major histocompatibility complex (MHC) driven immune responses present in more diverse human populations. This possibility is supported by work showing that the bobcat populations in the southeastern USA, where all of our *C. felis* samples were obtained, cluster into one subclade of the continental bobcat population when analyzed by either mitochondrial DNA (mtDNA) or microsatellite data [30].

Another possibility is that the observed lack of genetic polymorphism is due to a relatively recent population bottleneck, as has been postulated for some populations of *P. falciparum*, which it should be noted still maintain significantly greater levels of genetic diversity in AMA1 [31]. A final possible concern is that our sample size is too small to assess the variability of the *ama1* gene in infected populations of domestic cats and bobcats. Two lines of evidence suggest that this is not the case. First, in the comparison of nucleotide diversity between related apicomplexans, all sample sets, save that for *P. falciparum*, had significantly fewer samples, yet showed dramatically higher diversity suggesting sample size is not limiting our ability to detect diversity. Next, in previous work using the ITS regions we successfully characterized population structure in 88 samples from Arkansas and Georgia [9]. Whatever the cause, the remarkable sequence conservation observed in the *C. felis ama1* gene makes this a poor marker for exploring correlations between parasite genetics and clinical outcomes. Thus far, neither the now widely used rRNA internal transcribed spacer regions nor the *ama1* gene reported here have been adequate for fine-scale population genetic studies, nor for rigorous testing of genetic correlates of clinical outcome, highlighting the need for better molecular markers for the study of population genetics within *C. felis*.

## Conclusions

Our results support that the sequence of *C. felis ama1* gene is uniquely conserved compared to other related apicomplexans. While limiting its utility as a genetic marker for clinical outcome, the lack of polymorphism in *C. felis * AMA1 suggests that it may be an effective target for vaccine development for cytauxzoonosis.

## Methods

### Samples and DNA isolation

Ethylenediaminetetraacetic acid (EDTA) anticoagulated whole blood samples from 84 domestic cats with acute cytauxzoonosis were obtained from diagnostic laboratories and private veterinary hospitals in Arkansas (*n* = 53), Georgia (*n* = 30) and Florida (*n* = 1). EDTA whole blood samples from 9 bobcats originating from Arkansas (*n* = 2), Georgia (*n* = 1), Florida (*n* = 5) and California (*n* = 1) were obtained from the United States Department of Agriculture and the Southeastern Cooperative Wildlife Disease Study. Genomic DNA (gDNA) was purified from 200 µl of whole blood using the QIAmp DNA Blood Mini Kit (Qiagen Inc., Valencia, CA, USA). To confirm *C. felis* infection status and assess for the presence of inhibitors, real-time polymerase chain reaction (PCR) for the *C. felis 18S* rRNA gene and for the feline reference gene glyceraldehyde 3-phosphate dehydrogenase (GAPDH) was performed on purified gDNA samples using previously published methods [32, 33]. PCR reactions without gDNA template were included as a negative control.

### Isolation of *C. felis ama1* sequence by PCR

Using a homology based PCR approach, degenerate primers for *C. felis ama1* were designed with Geneious v9.1 using an alignment of *ama1* sequences from related apicomplexan parasites including *Plasmodium* spp., *Theileria* spp. and *Babesia* spp., to amplify a predicted PCR product of approximately 760 bp. PCR reactions were performed using the following reaction parameters: 25 pmol each of degenerate primer (5′-ATG GCN AAR TTY GAY ATH GC-3′ and 5′-GCC CAR TTD ATN CCN ACN CC-3′), 1× concentration of PCR Buffer II (Applied Biosystems, Foster City, CA, USA), 1.25 U of Taq Polymerase, 5 µl of gDNA template, 1.5 mM MgCl_2_, and 200 µM of each dNTP. Thermal cycling parameters included an initial denaturation at 95 °C for 5 min, followed by 36 amplification cycles (98 °C for 10 s, 56 °C for 20 s, and 68 °C for 90 s). PCR products were visualized by gel electrophoresis, purified using a QIAquick® PCR Purification Kit (Qiagen, Valencia, CA, USA), and sequenced bi-directionally *via* Sanger sequencing.

### PCR-based sequencing of a *C. felis ama1* gene region

PCR amplification and sequencing primers were derived from the amplicon sequence produced by the degenerate primers and designed with Geneious v9.1. PCR was performed on all samples using above described parameters and 25 pmol of each primer (5′-ACA CAT TAT TAT CGT ATG CCA-3′ and 5′-ACT AAA GTT ATT ATT CCT TAA CTC C-3′). PCR products were purified and sequenced bi-directionally to yield a 677 bp region for analysis. Histograms from all samples were analyzed to confirm presence or absence of nucleotide substitutions, insertions and/or deletions. Nucleotide and amino acid alignments of the *C. felis ama1* gene region from 84 domestic cats and 9 bobcats were performed using ClustalW software [34].

### Calculation of genetic diversity

Two measures of genetic diversity, nucleotide diversity (π) [35] and haplotype diversity (H) [36], were calculated using the DNA Polymorphism function of DnaSP v6.10.03. To directly compare homologous regions of AMA1 from related apicomplexans, an amino acid alignment of AMA1 sequences from *Theileria annulata*, *Babesia bovis*, *Babesia bigemina*, *Plasmodium falciparum* and *Plasmodium vivax* was constructed using Geneious v10.2 and MUSCLE v 3.8.425. Based upon this alignment, homologous nucleotide regions were determined from nucleotide alignments of *ama1* genes from each of the species and submitted to DnaSP for analysis.

## Abbreviations

AMA1: Apical membrane antigen-1 protein
*ama1*: Apical membrane antigen-1 gene
EDTA: Ethylenediaminetetraacetic acid
GAPDH: Glyceraldehyde 3-phosphate dehydrogenase
gDNA: Genomic DNA
H: Haplotype diversity
ITS: Internal transcribed spacer region of the ribosomal RNA operon
MHC: Major histocompatibility complex
mtDNA: Mitochondrial DNA
π: nucleotide diversity
PCR: Polymerase chain reaction
SNP: Single nucleotide polymorphism
